# Pharmacokinetics and Ex Vivo Activity of 7-Methylxanthine, an Inhibitor of Monosodium Urate Crystallization

**DOI:** 10.3390/biomedicines13061411

**Published:** 2025-06-09

**Authors:** Miguel D. Ferrer, Jaume Dietrich, Bernat Isern, Maria del Mar Pérez-Ferrer, Joan Albertí, Félix Grases, Antònia Costa-Bauzà

**Affiliations:** 1 Renal Lithiasis and Pathological Calcification Group (LiRCaP), Research Institute of Health Sciences (IUNICS), University of the Balearic Islands, 07122 Palma, Spain; miguel-david.ferrer@uib.es (M.D.F.); jaume.dietrich@uib.cat (J.D.); bernat.isern@uib.es (B.I.); mmar.perez@uib.es (M.d.M.P.-F.); fgrases@uib.es (F.G.); 2 Health Research Institute of the Balearic Islands (IdISBa), 07010 Palma, Spain; 3 Molecular Biology, Health Geography, and One Health (MolONE), University of the Balearic Islands, 07122 Palma, Spain; 4 ADMETRA Consulting, 07010 Palma, Spain; joan.alberti@admetra.com

**Keywords:** 7-methylxanthine, pharmacokinetics, oral administration, monosodium urate, gout

## Abstract

**Background/Objectives:** 7-Methylxanthine (7-MX) is a naturally occurring metabolite of caffeine and theobromine that can inhibit the crystallization of monosodium urate (MSU) and may be useful for the prevention or treatment of gout. However, the pharmacokinetics and ex vivo activity of 7-MX remain poorly characterized. **Methods**: The present study assessed the pharmacokinetics of 7-MX in Sprague Dawley rats following a single oral dose (30 mg/kg), and the ex vivo inhibition of MSU crystallization by 7-MX in rat plasma after the repeated administration of oral 7-MX. **Results**: The pharmacokinetic analysis showed that 7-MX reached peak plasma concentration (C_max_ ≈ 30 µM) at 30 min after administration (t_max_), the terminal half-life was approximately 1.4 h, and there was no evidence of accumulation after repeated daily dosing. After repeated administration, the relationship between dose (30 or 60 mg/kg) and plasma concentration was proportional. In vitro and ex vivo crystallization assays demonstrated that 7-MX inhibited MSU crystallization in a concentration-dependent manner. The in vitro studies showed that 100 µM 7-MX inhibited up to 74% of MSU crystallization under supersaturated conditions (400 mg/L urate). The ex vivo experiments indicated that plasma from rats that received 30 or 60 mg/kg of 7-MX had 41.4% and 52.6% inhibition of crystallization, consistent with the measured plasma concentrations. **Conclusions**: These findings confirm that oral administration of 7-MX to rats led to a plasma level that was sufficient to decrease MSU crystallization in plasma, and there were no observable toxicities. These results support the potential of 7-MX as a safe oral treatment for gout, especially in combination with urate-lowering therapies, such as allopurinol. Further clinical investigations are warranted to confirm the therapeutic potential of 7-MX in humans.

## 1. Introduction

7-Methylxanthine (7-MX) is a mono-methylxanthine that is rare in nature, but occurs as an intermediate in the biosynthesis of caffeine (1,3,7-trimethylxanthine) and dimethylxanthines (e.g., theobromine, 3,7-dimethylxanthine) by plants [1]. 7-MX also occurs as a metabolite in human plasma and urine after the consumption of caffeine or theobromine [2,3]. The metabolism of caffeine and theobromine includes different demethylation reactions that produce xanthines with decreased methylation [4].

In addition to its role as an intermediate in the synthesis of methylxanthines in plants [1] and as a metabolic product of caffeine and theobromine degradation in animals [4], 7-MX also functions in other biological processes. Thus, several studies demonstrated that 7-MX inhibited and slowed the progression of myopia in adolescents [5,6]. Our recent studies demonstrated that 7-MX inhibited uric acid (UA) crystallization in a solution that mimicked urine [7] and increased the solubility of monosodium urate (MSU) in solutions that mimicked synovial fluid [8,9] and urine [9]. The 7-MX-mediated increased solubility of urate occurs due to the formation of clusters (aggregates) of the urate ion and 7-MX, and these aggregates decrease the supersaturation of MSU and the thermodynamic driving force (ΔG) of crystallization. All of these molecules share a similar structure, and through hydrogen bonding and aromatic stacking interactions, aggregates are formed that decrease supersaturation [10,11]. Although it may appear that 7-MX increases the solubility of MSU, there is actually a decrease in its supersaturation due to formation of stable adducts between urate ions and 7-MX, analogous to the interaction between theobromine and UA [10,11]. 7-MX can prevent the crystallization of MSU for more than 7 days, and the necessary concentration of 7-MX depends on the concentration of urate in the medium: a higher concentration of urate requires a higher concentration of 7-MX [8,9]. These effects of 7-MX indicate its potential use for preventing the formation of MSU deposits in the joints, a hallmark of gout.

Importantly, 7-MX does not have toxic effects in experimental animals. In particular, studies of its chronic and subchronic toxicity, genotoxicity, and mutagenicity have concluded that an oral dose of 7-MX up to 1000 mg/kg body weight had no toxic effects [12,13,14]. In addition, clinical studies that examined the effectiveness of 7-MX in slowing the progression of myopia administered oral 7-MX to children at a doses of 1200 mg/day (3 doses of 400 mg every 8 h) and found no adverse effects [5].

Considering the possible therapeutic effects of 7-MX for the treatment or prevention of gout, it is necessary to establish its pharmacokinetic properties. The only data in humans indicated that an oral dose of 400 mg in adults led to a maximum serum concentration (C_max_) of about 20 μmol/L, and the terminal half-life (t_1/2_) was 200 min [6]. In rabbits, a single oral dose of 30 mg/kg 7-MX led to a C_max_ of about 70 μmol/L and a t_1/2_ of about 1 h [15]. However, there is very limited information about the relationship between the oral dose of 7-MX and its plasma concentration and half-life.

The aim of this study was to characterize the pharmacokinetics of 7-MX in Sprague Dawley rats, examine its possible accumulation during 8 consecutive days of administration, and determine its ex vivo activity in the inhibition of MSU crystallization in rat plasma.

## 2. Materials and Methods

### 2.1. Reagents, Solutions, and Animal Experiments

UA and 7-MX were purchased from Sigma-Aldrich (St Louis, MO, USA). Optima LC/MS-grade acetonitrile was obtained from Fisher Scientific (Loughborough, UK). All solutions were prepared in ultra-pure H_2_O from a Milli-Q system and passed through 0.45 μm filters. All animal procedures were conducted in accordance with protocols approved by the Animal Experimentation Ethics Committee (CEEA) of the University of the Balearic Islands (protocol number SSBA 06/2024 AEXP). Rats were fed with a standard diet for adult and maintenance animals (SAFE^®^ A40, SAFE, Augy, France).

### 2.2. Pharmacokinetics of 7-MX After a Single Dose

Single-dose experiments used 12 8-week-old Sprague Dawley rats (6 males with a mean body weight of 277 ± 8 g and 6 females with a mean body weight of 213 ± 17 g). One day before the intervention, both posterior legs of the animals were shaved. Then, the animals were given 30 mg/kg of 7-MX in 1% carboxymethylcellulose (CMC) by oral gavage. Blood was collected before and at different times after administration (0, 0.5, 1, 2, 3, 4, 6, 8, and 24 h) by puncture of the saphenous vein and collected in Microvette tubes with ethylenediaminetetraacetic acid (EDTA) as an anticoagulant. Samples were collected from 3 males and 3 females at each time to ensure that a maximum of four extractions (200 µL each) were collected over 24 h in each animal. The pharmacokinetic parameters were then calculated using Kinetica software version 5 (Alfasoft Limited, Luton, UK).

### 2.3. Ex Vivo Activity of 7-MX After Repeated Administration

The ex vivo activity of 7-MX was examined using 18 10-week-old Sprague Dawley rats (9 males with a mean body weight of 294 ± 8 g and 9 females with a mean body weight of 225 ± 13 g) over the course of 7 days. Three groups of rats were established, each with 3 males and 3 females. Group 1 received daily oral administration of 0.5% CMC; Group 2 received daily oral administration of 7-MX at 30 mg/kg in 0.5% CMC; and Group 3 received daily oral administration of 7-MX at 60 mg/kg in 0.5% CMC. On day 8, all animals received a final treatment and were sacrificed 30 min later (t_max_, based on single-dose studies) to determine the C_max_. Blood was obtained by exsanguination of the anesthetized animals (achieved by continuous inhalation of isoflurane using a vaporizer in an induction chamber with 3% oxygen and a flow rate of 1.5 L/min) by cardiac puncture. After exsanguination, the animals were euthanized by administering an overdose of isoflurane.

### 2.4. Quantification of 7-MX in Plasma

For determination of the plasma concentration of 7-MX, ultra-high-performance liquid chromatography with tandem mass spectrometry (a Thermo Scientific Dionex Ultimate 3000 coupled with a Thermo Scientific QExactive-Orbitrap Ms, Thermo Fisher, Dreieich, Germany) was performed. The method was based on a previous method developed for the determination of xanthines in urine [16].

Because plasma contains high concentrations of biomolecules that can cause baseline noise, the samples were deproteinized before injection. Thus, the plasma samples were treated with 1:1 *v*/*v* acetonitrile LC/MS grade [17], vortexed, and then centrifuged at 14,500 rpm for 10 min. The supernatant was collected and diluted with a solution of 1% formic acid in Milli-Q water.

A calibration curve was established using 7-MX standards (0, 0.2, 0.5, 1, 2, 5, 10, 25, 50, 75, and 100 ng/mL). For each measurement, the corresponding quantity of deproteinized plasma from rats that did not receive 7-MX was used with the standards for matrix-match analysis. Each sample was analyzed in triplicate, and the limit of quantification was 0.2 ng/mL.

The mobile phases consisted of formic acid 1% in Milli-Q water (A) and formic acid 1% in acetonitrile (B).

For the analysis of pharmacokinetics after a single dose, the plasma dilution (1:250) was obtained by diluting 40 µL of the supernatant in 4.96 mL of 1% formic acid in Milli-Q water.

For the analysis of samples obtained at C_max_ after repeated administration, the plasma dilution (1:500) was obtained by diluting 20 µL of the deproteinized plasma in 4.98 mL of 1% formic acid in Milli-Q water. This higher dilution was used because the concentrations detected in the single-dose experiment indicated this was sufficient to allow reliable detection at a higher dilution, and led to decreased baseline noise and greater precision.

This method showed the recovery of >94% of 7-MX on the deproteinized samples, along with precision of >96% and accuracy of >95%.

### 2.5. In Vitro Crystallization of MSU in Rat Plasma

Experiments that examined the crystallization of MSU in rat plasma were carried out in 96-well plates (Brand GMBH, Wertheim, Germany) by adapting a previous method used for measuring crystallization in human plasma [9]. Rat plasma was centrifuged at 10,000 rpm for 10 min prior to preparation of the plate, and the supernatant was then used as the crystallization medium.

#### 2.5.1. Optimization of the Method

Adaptation of the method used for human plasma was necessary because rat plasma did not crystallize MSU using these previous conditions. The optimization procedure consisted of a crystallization assay in which urate was added to plasma to achieve final concentrations of 250, 300, 350, 400, and 500 mg/L. Because none of these concentrations led to crystallization, sodium chloride was added (final concentration: 200 mM) because a low ion concentration can prevent crystallization. Therefore, the method used to induce MSU crystallization in rat plasma and evaluate the inhibitory activity of 7-MX used 300 or 400 mg/L of urate with 200 mM of NaCl.

#### 2.5.2. In Vitro Inhibition of MSU Crystallization in Plasma

After optimization, MSU crystallization assays were carried out in 96-well plates by adding 300 or 400 mg/L of urate and 200 mM of NaCl to rat plasma with 0, 3, 10, 25, 50, 75, or 100 µM of 7-MX.

Thus, 246.7 µL of rat plasma, 23.3 µL of Milli-Q water, and 20 µL of 3.5 M NaCl were added to two wells. Then, 60 µL of 1.75 g/L urate was added to one well to achieve a concentration of 300 mg/L, and 60 µL of 2.33 g/L urate was added to the other well to achieve a concentration of 400 mg/L. The initial urate concentration in each well was quantified using a uricase kit (Spinreact, Girona, Spain).

For preparation of the plate, 28 wells (7 columns, 4 wells per column) were filled with 246.7 µL/well of the centrifuged plasma. Rows 1 and 2 of were used as the two replicates for the assays with 300 mg/L UA, and rows 3 and 4 for the assays with 400 mg/L UA. Then, 60 µL of 1.75 g/L UA was added to the wells in rows 1 and 2 of each column, and 60 µL of 2.33 g/L UA was added to the wells in rows 3 and 4 of each column. The seven columns were used for assays with 0, 3, 10, 25, 50, 75, and 100 µM 7-MX. Thus, 23.3 µL of Milli-Q water or 23.3 µL of different concentrations of 7-MX (0.045, 0.150, 0.376, 0.751, 1.127, or 1.5 mM) were added to the wells of columns 1 to 7 to obtain the desired concentrations. Finally, 20 µL of 3.5 M NaCl was added to each of the 28 wells. The plate was then sealed and incubated at 32 °C for 96 h. Then, the amount of urate in solution was determined by the uricase method after appropriate dilution. The amount of crystallized MSU (expressed as urate ion) was obtained by subtracting the urate remaining in solution from the initial urate concentration (before incubation).

The inhibition of MSU crystallization was calculated by comparing the amount of crystallized MSU in the control sample (blank) with that in the sample containing 7-MX:

$$\%\;Inhibition\;of\;crystallization=\frac{Crystallized\;urate\;\left(blank\;plasma\right)-Crystallized\;urate\;(7-methylxanthine)}{Crystallized\;urate\;(blank\;plasma)}\times 100$$

A concentration–response curve was generated using a nonlinear regression model (variable slope, four parameters) using GraphPad Prism version 10 (GraphPad Software, La Jolla, CA, USA).

#### 2.5.3. Ex Vivo Inhibition of MSU Crystallization in Plasma

The MSU crystallization assay was also performed using plasma obtained from rats that received 7-MX. As in the in vitro experiments, plasma samples were centrifuged at 10,000 rpm for 10 min before this assay.

For assays with 300 mg/L of urate, 620 µL of centrifuged plasma was added to a 1.5 mL Eppendorf tube with 45.93 µL of 3.5 M NaCl and 137.78 µL of 1.75 g/L urate; for assays with 400 mg/L of urate, the solution had 2.33 g/L urate. A total of 54 µL from each Eppendorf tube was used for quantification of the initial urate concentration, and the remaining plasma was added to wells (350 µL each). The plate was then sealed and incubated at 31 °C for 96 h. Then, the plate was removed, and the amount of urate remaining in solution was determined by the uricase method after appropriate dilution. The amount of crystallized MSU (expressed as urate ion) was obtained by subtracting the urate remaining in solution from the initial urate concentration (before incubation).

The results obtained in male and female rats were pooled after a two-way ANOVA confirmed that sex had no effect on the inhibition of MSU crystallization (GraphPad Prism version 10.0). The results from rats that received different doses of 7-MX were analyzed using one-way ANOVA with Tukey’s post hoc test. Pearson’s correlation analysis was used to assess the correlation between the circulating level of 7-MX and the inhibition of MSU crystallization. A *p*-value below 0.05 was considered significant.

## 3. Results

### 3.1. Oral Pharmacokinetics of 7-MX

**Figure 1:**
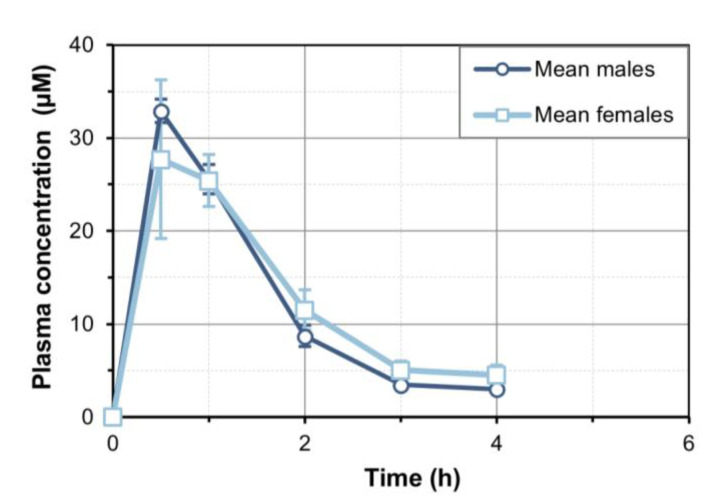
Plasma concentration of 7-MX in male and female Sprague Dawley rats after single oral dose (30 mg/kg). Concentrations were quantified in plasma samples from 6 males and 6 females. Results are expressed as mean ± SD (N = 3 per sex and time point).

We administered a single oral dose of 30 mg/kg 7-MX to rats, and then collected plasma. Samples obtained between 0 and 4 h were used for statistical fitting using Kinetica software. There was no detectable 7-MX at 6 h. The results show that oral 7-MX had a t_max_ at 30 min (Figure 1), and the statistical fitting indicated that the C_max_ was 32.9 µM in males and 27.7 µM in females, the AUC_0→t_ was 49.4 µmol·h/L in males and 51.8 µmol·h/L in females, and the t_1/2_ was 1.31 h in males and 1.48 h in females (Table 1).

We then determined if the repeated administration of 7-MX (once daily for 7 days) led to a steady level or an accumulation of 7-MX. Thus, we measured the level at the tmax (30 min) after the final administration of 7-MX (Table 2). The results show that the circulating level at this time was in the same range as that obtained after a single dose of 30 mg/kg and that the level increased by about 1.8-fold when the dose was doubled to 60 mg/kg. These results indicate no accumulation of 7-MX after repeated daily dosing, presumably because of the short in vivo half-life of this compound (<2 h).

### 3.2. Inhibition of MSU Crystallization

#### 3.2.1. In Vitro Crystallization Assays

**Figure 2:**
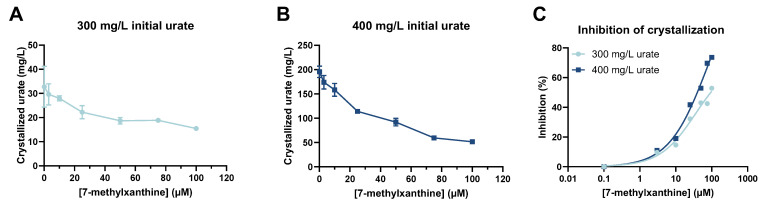
Effect of 7-MX concentration in rat plasma on amount of MSU crystallization when initial urate concentration was 300 mg/L (**A**) and 400 mg/L (**B**), and percent inhibition of MSU crystallization by different concentrations of 7-MX for urate concentrations of 300 and 400 mg/L (**C**). Results in (**A**,**B**) are means ± SDs (N = 2). Curves in (**C**) were from fits to nonlinear regression model with four parameters.

The results of our in vitro MSU crystallization assays showed that the amount of crystallized MSU (mg/L urate) decreased as the concentration of 7-MX increased (Figure 2A,B). For an initial urate concentration of 300 mg/L, the amount of crystallized urate in the absence of 7-MX was 32.8 mg/L (10.9% of total urate), and the amount was 15.5 mg/L (5.2% of total urate) in the presence of 100 µM 7-MX. With an initial urate concentration of 400 mg/L, the amount of crystallized MSU in the absence of 7-MX was 195.6 mg/L (48.9% of total urate), and the amount was 51.5 mg/L (12.9% of total urate) in the presence of 100 µM 7-MX. Thus, the inhibitory effect of 7-MX was greater when the initial urate concentration was higher (Figure 2C).

#### 3.2.2. Ex Vivo Crystallization Assays

**Figure 3:**
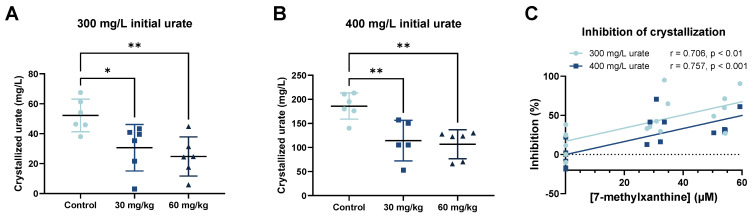
Effect of oral administration of 7-MX (0, 30, or 60 mg/kg) to Sprague Dawley rats on ex vivo inhibition of MSU crystallization in plasma after addition of 300 mg/L (**A**) and 400 mg/L urate (**B**), and percent inhibition of crystallization by different concentrations of 7-MX for urate concentrations of 300 and 400 mg/L (**C**). Results in (**A**,**B**) are means ± SDs (N = 6). * *p* < 0.05; ** *p* < 0.01. In (**C**), abscissa indicates plasma levels of 7-MX in rats that received 0, 30, or 60 mg/L 7-MX, and lines are from Pearson’s correlation. Dashed line represents an inhibition of 0%.

We also performed ex vivo crystallization assays of MSU using 300 or 400 mg/L urate and the plasma of the rats that received 0, 30, or 60 mg/kg of oral 7-MX (Figure 3A,B). For both levels of urate, crystallization decreased as the oral dose of 7-MX increased, similar to our in vitro crystallization results. For an initial urate concentration of 300 mg/L, the mean level of crystallized MSU was 52.2 mg/L in the absence of 7-methylxanthine, 30.6 mg/L (41.4% inhibition) in the presence of 24.2 µM 7-MX (corresponding to an oral dose of 30 mg/kg), and 24.8 mg/L (52.6% inhibition) in the presence of 43.4 µM 7-MX (corresponding to an oral dose of 60 mg/kg). For an initial urate concentration of 400 mg/L, the mean level of crystallized MSU was 186 mg/L in the absence of 7-methylxanthine, 114.2 mg/L (38.6% inhibition) in the presence of 24.2 µM 7-MX (corresponding to an oral dose of 30 mg/kg) and 106.7 mg/L (42.6% inhibition) in the presence of 43.4 µM 7-MX (corresponding to an oral dose of 60 mg/kg). Thus, the ex vivo inhibition of MSU crystallization had a significant and positive correlation with the concentration of plasma 7-MX (Figure 3C).

## 4. Discussion

This study is the first to describe the pharmacokinetics of 7-MX after oral administration to rats, and to document the effect of 7-MX on the inhibition of MSU crystallization in rat plasma.

Cytochrome P450 1A2 catalyzes the conversion of caffeine and theobromine into 7-MX [18], and some studies have examined the acute and chronic toxicity of 7-MX when administered to rats. One study that examined sub-acute oral toxicity administered 7-MX to rats at doses of 250 to 1000 mg/kg and reported no adverse effects [12]. Another study examined the effect of the long-term administration of 7-MX (90 days) and reported no mortality in rats treated with 250, 500, or 1000 mg/kg of 7-MX; however, the mortality rate was 10 to 40% in response to LD_50_ doses of caffeine (151–174 mg/kg) and theobromine (250 mg/kg) [13]. These toxicity studies revealed that oral 7-MX is safe in rats at a dose of up to 1000 mg/kg/day. Aside from these two reports, no other studies have examined the pharmacokinetics of 7-MX in rats or humans.

When we administered oral 7-MX at 30 mg/kg, a plasma C_max_ of approximately 30 µM was reached after about 30 min, the t_1/2_ was approximately 1.4 h, and 7-MX was undetectable after 6 h. We also found no differences in the pharmacokinetic profiles of male and female rats. When we administered oral 7-MX for 7 days, there was no evidence of accumulation, in accordance with the short in vivo half-life of this compound. We also observed dose-proportionality in terms of C_max_ when the oral dose was increased from 30 to 60 mg/kg. A previous study of 7-MX in rabbits reported a C_max_ of 70 µM and a t_1/2_ of about 1 h after a single oral dose of 30 mg/kg [6,15], in line with our results in rats. Despite the limited information about the pharmacokinetics of 7-MX in rats, there have been pharmacokinetic and metabolic studies of theobromine. Oral theobromine is rapidly and almost completely absorbed in rats [19] and humans [20,21,22], and seems to have greater bioavailability than oral 7-MX. Thus, an oral dose of 50 mg/kg theobromine had a C_max_ of 80 µg/mL at 18 min in Sprague Dawley rats [19]; our results showed that an oral dose of 30 and 60 mg/kg of 7-MX led to C_max_ values of approximately 5 and 10 µg/mL, respectively. However, it must be noted that our C_max_ values might have been slightly underestimated because our first sample was collected at 30 min, and the t_max_ for theobromine was reported to be between 18 and 36 min. However, the very large difference in the C_max_ and AUC values of these two compounds (692 µg·h/L for 50 mg/kg theobromine vs. 8.3 µg·h/L for 30 mg/kg 7-MX) confirms that the oral bioavailability is lower for 7-MX than theobromine. Approximately 10% of the total theobromine in an oral dose is transformed endogenously to 7-MX. Then, 7-MX can be directly excreted in urine or further metabolized to 7-methyluric acid before excretion [4]. However, doses of theobromine above 100 mg/kg are toxic, but there are no signs of 7-MX toxicity for doses up to 1000 mg/kg. For this reason, the direct administration of 7-MX rather than theobromine seems to be a better option to achieve a therapeutic plasma concentration of 7-MX.

As mentioned in the Materials and Methods, we had to modify the experimental conditions that were used to assess the in vitro inhibition of MSU crystallization because rat plasma has very low levels of urate. Thus, when using human plasma, 50 and 200 mg/L of urate induced MSU crystallization [9]; 50 µM 7-MX prevented MSU crystallization when the urate plasma concentration was 87 mg/L (addition of 50 mg/L); and 100 µM 7-MX prevented MSU crystallization when the urate concentration was 237 mg/L (addition of 200 mg/L) [9]. In contrast to this human assay, the addition of 300 or 400 mg/L urate with a final sodium concentration of 200 mM was required to induce MSU crystallization in rat plasma. Thus, a higher concentration of 7-MX was needed to prevent urate crystallization in rat plasma.

In view of our results regarding the in vitro inhibition of MSU crystallization, a level of about 30 µM 7-MX inhibited MSU crystallization by about 50%. For this reason, we gave rats 30 or 60 mg/kg of oral 7-MX and collected blood samples 30 min later, when levels of about 30 and 60 µM were expected.

Our ex vivo assay showed that the circulating level of 7-MX after oral administration was sufficient to inhibit MSU crystallization by 40% to 60%, depending on the dose and the initial concentration of urate. This confirms that a therapeutic level of 7-MX can be achieved by oral administration, and that the efficacy is proportional to the dose. These results are in agreement with those obtained when using human plasma, because the supersaturation of MSU in our assay with rat plasma was much higher than that for human plasma. In particular, previous research showed that when the human plasma concentration of urate was 237 mg/L, MSU crystallization was 64.25 mg/L in the absence of 7-MX and was 34.85 mg/L in the presence of 50 µM of 7-MX [9]. In our study of rat plasma, when the urate concentration was 300 mg/L, the level of crystallized urate was 52.2 mg/L in the absence of 7-MX and was 24.8 mg/L in the presence of 43.4 µM 7-MX. Thus, the plasma concentration of 7-MX needed to inhibit MSU crystallization (30–100 µM) is attainable with doses that are safe in animals [13] and humans [6].

It is important to consider the design of our experiments when interpreting the possible clinical relevance of our results. The test we used required some sodium urate crystallization to occur, thus implying high supersaturation of urate (300 or 400 mg/L), and also required the addition of sodium (200 mM). In humans, patients with gout typically have a plasma urate concentration around 60 mg/L and the typical concentration of sodium is about 150 mM. However, the inhibitory effect of 7-MX is greater at a lower urate concentration [8], as confirmed in our ex vivo assay (Figure 3C). Thus, in human plasma, which has lower supersaturation conditions, 7-MX will be more effective in inhibiting MSU crystallization, so a lower dose may be sufficient.

Finally, our results suggest that the combination of 7-MX with allopurinol may be an effective approach to gout treatment. Allopurinol inhibits xanthine oxidoreductase, the enzyme in the purine catabolism pathway that converts hypoxanthine into xanthine and xanthine into UA. The combination of 7-MX with allopurinol may allow the use of lower doses of allopurinol, thus reducing the risk of its adverse effects (pruritus and rash).

Although the results of this research are promising and may contribute to the development of novel therapeutic strategies for gout, certain limitations in the experimental design should be acknowledged. These include the use of non-physiological conditions—specifically, an excess of urate and sodium—to induce monosodium urate crystallization in rat plasma, which may limit the direct translatability of the findings to clinical settings. Additionally, the limited number of replicates in the in vitro study is a constraint. Furthermore, interspecies differences in the pharmacokinetics and pharmacodynamics of 7-methylxanthine must be carefully considered before extrapolating these results to humans.

In conclusion, our results show that the oral administration of 7-MX to rats led to a plasma C_max_ that was proportional to the dose. The half-life of 7-MX was approximately 1.4 h, and there was no evidence of accumulation after repeated dosing and no differences in the pharmacokinetics of males and females. A level of 7-MX that can inhibit MSU crystallization was achieved by oral administration, and the efficacy of 7-MX was proportional to its dose. It is necessary to determine the effects of 7-MX on urate crystallization in humans and evaluate the potential clinical use of 7-MX.

## Figures and Tables

**Table 1:** The mean values of pharmacokinetic parameters in male and female Sprague Dawley rats after a single oral dose of 7-MX (30 mg/kg) (N = 3 per sex).

	Males	Females	Both Sexes
C_max_ (µM)	32.9	27.7	30.3
t_max_ (h)	0.5	0.5	0.5
t_1/2_ (h)	1.31	1.48	1.40
AUC_0→t_ (µmol·h/L)	49.4	51.8	50.6
AUC_inf_ (µmol·h/L)	55.1	61.4	58.2
MRT_0→∞_ (h)	1.72	2.15	1.95
Vss/F (L/kg)	5.7	6.3	6.0
Cl/F (L/h/kg)	3.28	2.94	3.10

C_max_: maximum concentration; t_max_: time of maximum concentration; t_1/2_: terminal half-life; AUC: area under the curve; MRT: mean residence time; Vss/F: volume of distribution in the steady state (oral administration); Cl/F: plasma clearance (oral administration); F: absolute oral bioavailability (unknown).

**Table 2:** Circulating levels of 7-MX at t_max_ (30 min) after daily administration of different doses (0, 30, 60 mg/kg) for 7 days to male and female Sprague Dawley rats.

7-MX Dose	Males 7-MX (µM)	Females 7-MX (µM)	Both Sexes 7-MX (µM)
0 mg/kg	n.d.	n.d.	n.d.
30 mg/kg	33.1 ± 2.0	29.6 ± 2.4	31.3 ± 2.8
60 mg/kg	59.7 ± 5.5	52.8 ± 2.1	56.3 ± 5.3

n.d.: not detectable; t_max_: time of maximum concentration in circulation (30 min after administration).

## Data Availability

The data presented in this study are available upon request from the corresponding author.

## Abbreviations

The following abbreviations are used in this manuscript:

7-MX	7-Methylxanthine
AUC	Area under the curve
Cl	Plasma clearance
C_max_	Maximum concentration
CMC	Carboxymethylcellulose
EDTA	Ethylenediaminetetraacetic acid
F	Absolute oral bioavailability
MRT	Mean residence time
MSU	Monosodium urate
SD	Standard deviation
t_1/2_	Terminal half-life
t_max_	Time of maximum concentration
UA	Uric acid
UHPLC-MS	Ultra-high-performance liquid chromatography with tandem mass spectrometry
Vss	Volume of distribution in the steady state
